# Biology of IL-36 Signaling and Its Role in Systemic Inflammatory Diseases

**DOI:** 10.3389/fimmu.2019.02532

**Published:** 2019-10-31

**Authors:** Zhi-Chao Yuan, Wang-Dong Xu, Xiao-Yan Liu, Xing-You Liu, An-Fang Huang, Lin-Chong Su

**Affiliations:** ^1^Department of Evidence-Based Medicine, School of Public Health, Southwest Medical University, Luzhou, China; ^2^Department of Nutrition and Food Hygiene, School of Public Health, Southwest Medical University, Luzhou, China; ^3^School of Traditional Chinese Medicine, Southwest Medical University, Luzhou, China; ^4^Department of Rheumatology and Immunology, Affiliated Hospital of Southwest Medical University, Luzhou, China; ^5^Department of Rheumatology and Immunology, Minda Hospital of Hubei Minzu University, Enshi, China

**Keywords:** IL-36, autoimmunity, immune cell, inflammation, cytokine

## Abstract

Interleukin (IL)-36 is a member of the IL-1 superfamily and includes three agonists (IL-36α, IL-36β, and IL-36γ) and an antagonist (IL-36Ra). IL-36 agonists bind to heterodimeric receptor complexes. Then, the heterotrimer complexes signal via intracellular functional domains, binding to downstream signaling proteins and inducing inflammatory responses. In this review, we summarized the current knowledge about the biological role of IL-36 and its correlation with systemic inflammatory diseases. The information collected will help to increase the understanding of the potential of IL-36 and may give clues for developing novel therapeutic strategies.

## Introduction

**Figure 1:**
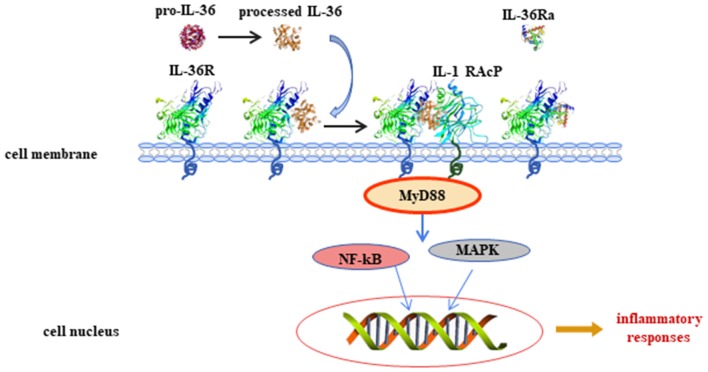
Interleukin (IL)-36 signal pathways induce inflammatory responses. Pro-IL-36 cytokines are inactive proteins and require post-translational processing to fully unleash the pro-inflammatory activity. IL-36 agonists bind to heterodimeric receptor complexes, including IL-36 receptor (IL-36R) and co-receptor IL-1 receptor accessory protein (IL-1RAcP). Subsequently, the heterotrimer complex binds to adaptor protein myeloid differentiated protein 88 (MyD88), activating mitogen-activated protein kinase (MAPK) and nuclear transcription factor kappa B (NF-κB) signaling cascade pathways, and regulates expression of target genes.

Interleukin (IL)-36 is an inflammatory cytokine and is a member of the IL-1 superfamily. IL-36 is composed of three agonists, IL-36α, IL-36β, and IL-36γ (previously called IL-1F6, IL-1F8, and IL-1F9) and an antagonist, IL-36 receptor antagonist (IL-36Ra, formerly known as IL-1F5). This terminology was unified into its current name in 2010 (1). The genes that encode for the IL-36 family of proteins are located on human chromosome 2 (2, 3). IL-36Ra is encoded by gene *IL-36RN*. IL-36Ra and IL-1Ra have 52% homologous amino acid sequence, and both function as receptor antagonists. IL-36 proteins are widely expressed in T cells, keratinocytes, and skin, lung, and gut cells. IL-36 agonists bind to receptors [IL-36R and IL-1 receptor accessory protein (IL-1RAcP)] and then activate the adaptor protein myeloid differentiated protein 88 (MyD88), mitogen-activated protein kinase (MAPK), and nuclear factor-kappa B (NF*-*κB) signaling pathways. Finally, these pathways initiate the regulation of target genes (4) (Figure 1). IL-36 is involved in immune cell activation, antigen presentation, and pro-inflammatory factor production. In recent years, IL-36 has attracted great interest because of its dysregulation in inflammatory diseases. The present study summarized information about the biology of IL-36 and the importance of IL-36 in common and polygenic inflammatory disorders, such as psoriasis, systemic lupus erythematosus (SLE), rheumatoid arthritis (RA), and inflammatory bowel disease (IBD).

### Processing of IL-36

IL-36 cytokines are inactive as full-length proteins and require post-translational processing to unleash their pro-inflammatory activity. Evidence has suggested that neutrophil-derived cathepsin G (Cat G), elastase, and proteinase-3 affect the processing and activation of IL-36α, IL-36β, and IL-36γ. Incubation of the three IL-36 cytokines with activated neutrophil supernatants results in robust activation of these proteins, suggesting that soluble neutrophil proteases released during degranulation can activate IL-36 cytokines (5). For instance, incubation with either Cat G or elastase induces IL-36α processing and activation by cleavage at lysine 3 (Lys3) and alanine 4 (Ala4), respectively (6). IL-36β was selectively stimulated by Cat G through its cleavage at residue arginine 5 (Arg5). IL-36γ was robustly activated by elastase or proteinase-3 by means of cleavage at the residue valine 15 (Val15) (6). In human primary dermal fibroblasts, keratinocytes, and skin equivalents, IL-36Ra is cleaved into an active form by neutrophil-derived protease elastase (7). Cathepsin S (Cat S) was the major IL-36γ-activating protease expressed by barrier tissues, and Cat S functions by cleaving IL-36γ between residues glutamic acid 17 (Glut17) and serine 18 (Ser18) (8). The processing of IL-36 is similar to that of other members of the IL-1 family, such as IL-37 and IL-1β. For example, intracellular pro-IL-37 is activated by caspase-1, and mature IL-37 translocates into the nucleus to inhibit the transcription of pro-inflammatory genes. Extracellular pro-IL-37 was affected by proteases to play an anti-inflammatory role (9). Pro-IL-1β undergoes proteolytic cleavage by caspase-1 to be processed into its active form, pro-inflammatory cytokine IL-1β (10).

### Biological Functions of IL-36

IL-36 plays a role in different cell types as shown in human and animal models via binding to IL-36R. IL-36R transcripts cannot be detected in CD4^+^ or CD8^+^ T cells or in neutrophils isolated from normal mouse blood (11). Monocytes, myeloid dendritic cells (mDCs), and monocyte-derived dendritic cells (MDDCs) from normal mice expressed IL-36R and responded to IL-36, and the mDCs' surface expressed more IL-36R than that expressed on monocytes. In skin keratinocytes of normal mice and human, IL-36R was abundantly expressed (11). The role of IL-36 in keratinocytes has been widely discussed in recent years. Cat G-processed IL-36β is expressed in primary human keratinocytes. The active IL-36β induced the expression of inflammatory cytokines and chemokines from keratinocytes, including IL-17C, granulocyte colony-stimulating factor (G-CSF), IL-8, chemokine (C-X-C motif) ligand 1 (CXCL-1), and chemokine (C-C motif) ligand 20 (CCL-20) (6). IL-36β also induced IL-17A and tumor necrosis factor (TNF) expression in human keratinocytes, which could be synergized by IL-22 (12). Interestingly, IL-36 synergized with IL-17A and TNF-α to increase the expression of IL-6, IL-8, and TNF-α in primary human keratinocytes (13). Normal human keratinocytes stimulated with IL-36α, IL-36β, or IL-36γ produced increased levels of CCL3, CCL4, CCL20, CCL5, CXCL8, and CCL20, demonstrating that, following IL-36 exposure, keratinocytes are potent sources of macrophage, T cell, and neutrophil chemokines (11). When keratinocytes from healthy donors were cultured with IL-36α, IL-36β, or IL-36γ, the expression of IL-17A signaling-related genes (*IL36G, S100A7*, and *LCN2*), p38-MAPK signaling-related genes (*IRAK2* and *PLA2G4D*), and granulocyte/agranulocyte adhesion and diapedesis [*CCL20, IL-8*, and *matrix metallopeptidase 9* (*MMP9*)] were upregulated, suggesting that IL-36 cytokines are able to amplify keratinocyte inflammatory responses by promoting not only their own expression but also that of molecules related to Th17 signaling (14). Levels of genes encoding interferon (IFN) receptor components, such as IFN gamma receptor 1 (*IFNGR1*), *IFNGR2*, and *IFNAR2*, were increased by IL-36 (IL-36α, IL-36β, and IL-36γ) stimulation in human primary epidermal keratinocytes (15). Interestingly, the expression of genes with two or more IFN regulatory factor 1 (IRF1) binding sites was upregulated by IL-36 family stimulation (15). IκBζ is an atypical IκB (inhibitor of NF-κB) member, and it is a specific transcriptional regulator for *NF*-κ*B* target genes. IκBζ expression was induced when human skin epidermal keratinocytes were exposed to IL-36 (16). It is notable that IL-36-mediated induction of IκBζ was required for the expression of downstream genes involved in inflammatory signaling, neutrophil chemotaxis, and leukocyte activation (16). In addition, IL-36γ-stimulated human endothelial cells promoted the generation of IL-8, CCL2, CCL20, and adhesion molecules [vascular cell adhesion molecule-1 (VCAM-1) and intercellular adhesion molecule (ICAM-1)] was upregulated in IL-36γ-treated endothelial cells (17). Therefore, IL-36 may regulate keratinocyte- and endothelial cell-mediated inflammatory response.

Human monocytes cultured with IL-36 were activated, and IL-36 stimulation significantly upregulated expression of IL-1α, IL-1β, and IL-6 (11). In murine dendritic cells (DCs), IL-36 agonist treatment upregulated activation markers of DCs, such as CD80, CD86, and MHCII, and it induced the production of IL-6 and IL-12 (18). When murine MDDCs were stimulated with IL-36β, the levels of IL-12p70, IL-23, and IL-10 became elevated (19). Furthermore, in IL-36α knockout (–/–) mice, the number of neutrophils recruited to the epidermis and dermis was reduced, and CXCL1 generation was downregulated (20). Together, the above findings indicate that IL-36 plays an important role in innate immune response.

**Figure 2:**
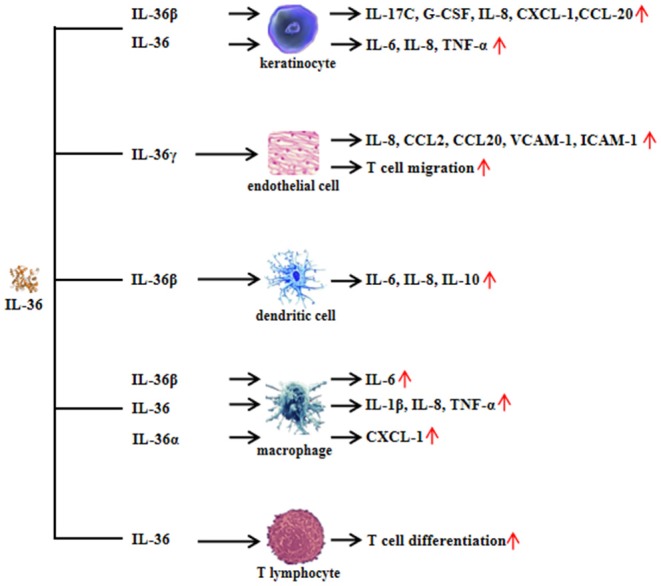
Functional role of interleukin (IL)-36 in non-immune cells and immune cells. Non-immune cells include keratinocytes and endothelial cells. Immune cells include dendritic cells, macrophages, and T lymphocytes.

When CD4^+^ T cells from IL-36R^−/−^ mice were co-cultured with IL-36γ, regulatory T (Treg) cell differentiation was inhibited compared with CD4^+^ T cells from wild-type mice co-cultured with IL-36γ (21). When isolated CD4^+^ T cells from MyD88^−/−^ mice or p50^−/−^ mice are co-cultured with IL-36γ, the differentiation of Treg cells increased (21). In contrast, abrogation of IL-36γ-induced IL-9 production was observed in CD4^+^ T cells from MyD88^−/−^ or p50^−/−^ mice when stimulated with IL-36γ. These findings showed that IL-36γ may inhibit Treg cell differentiation and promote Th9 cell differentiation by downstream signaling pathways, including MyD88 and NF-κB (21). CD4^+^ T cells stimulated with IL-36α under Th1 polarizing conditions showed that IL-36α potently drove Th1 responses (22). IL-36β upregulates the production of IL-18 and IL-12p70 in MDDCs, suggesting the induction of a Th1 phenotype (23). These studies illustrated that IL-36 is important in effector T-cell differentiation (Figure 2).

### Regulation of IL-36

**Figure 3:**
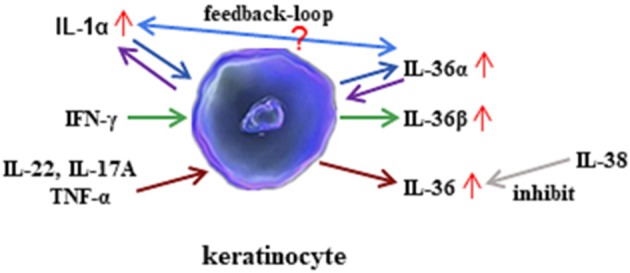
Regulation of interleukin (IL)-36 in keratinocytes. IL-36α regulates the generation of IL-1α in keratinocytes. In turn, IL-1α affects the synthesis of IL-36α in keratinocytes. However, the mechanism of feedback loop needs to be elucidated in the future. Interferon (IFN)-γ induces IL-36β production in keratinocytes. In addition, inflammatory cytokines such as IL-22, IL-17A, and tumor necrosis factor (TNF)-α induce all three agonists' expression, whereas the effect may be inhibited by IL-38 administration.

Available evidence has suggested that IL-36 regulates the function of both non-immune cells and immune cells. IL-36 can also be regulated by different inflammatory components and cells. In mouse keratinocytes, IL-1α induced IL-36α expression, and the levels of IL-36α from inflamed IL-1R1^−/−^ skin was significantly lower than those of wild-type mice (11). Therefore, IL-1α is an important regulator of IL-36α expression. In return, IL-36α may regulate IL-1α in a feedback loop, where primary mice keratinocytes rapidly induced IL-1α in response to IL-36α stimulation. Interestingly, the induction of IL-1α correlated with increased IL-36α expression (11). Moreover, the levels of IL-1α released from imiquimod-treated skin were significantly lower in the absence of IL-36α than in the presence of IL-36α (11). Therefore, this result suggested that IL-36α may induce IL-1α expression. In human keratinocytes, IL-22, IL-17A, and TNF-α induce the production of all three IL-36 subfamilies, and IFN-γ selectively induces IL-36β production (13). With macrophage-activating lipopeptide 2 (MALP-2) stimulation, IL-36α expression was highly enhanced in human primary keratinocytes (24). The double-stranded RNA analog poly(I:C) induces pyroptosis in human keratinocytes, thereby facilitating the extracellular release of IL-36γ, whereas suppression of caspase-3/7 and caspase-1 blocks the release of IL-36γ from poly(I:C)-treated cells (25). IL-38 is known to function as an antagonist of IL-36R (26). IL-38 binds to IL-36R and IL-1RAcP, inhibiting the biologic function of IL-36 (19) (Figure 3).

### Role of IL-36 in Systemic and Inflammatory Diseases

#### IL-36 in SLE

SLE is an autoimmune disease characterized by damage to multiple systems and organs (such as kidney, bones, and skin) and dysregulated autoantibody production. Studies have shown that IL-36 expression and function may correlate with the pathogenesis of SLE. In the serum and tubuli of nephritic kidney biopsies of SLE patients, IL-36 expression (IL-36α and IL-36γ) was significantly higher than that in healthy controls (27). Elevated expression of IL-36α and IL-36γ positively correlated with the SLE disease activity index. In contrast, serum levels of IL-36Ra were significantly lower in SLE patients than in healthy controls (28–30). IL-36 stimulation promoted the production of inflammatory cytokines that participate in the development of SLE, including IFN-γ, TNF-α, IL-4, IL-17, IL-6, and IL-8 (30). Evidence suggested that regulatory B cell (Breg) subsets, such as CD19^+^CD24^high^CD27^+^ Bregs, could downregulate adaptive/innate immune responses (28), and the proportion of CD19^+^CD24^high^CD27^+^ cells in total B lymphocytes negatively correlated with the proportion of IL-36R-positive B lymphocytes. It is hypothesized that there may be an inverse correlation between circulating inflammatory IL-36 and anti-inflammatory Breg subpopulations. However, how IL-36 regulates this specific subset and, therefore, contributes to SLE pathogenesis needs further investigation. In addition, in our previous studies, Th17 cell-related cytokines, such as IL-17 and IL-21, have been found to contribute to SLE pathogenesis (31, 32). Interestingly, IL-17^−/−^ mice did not develop lupus after pristane injection, whereas wild-type mice developed lupus (33), as evidenced by increased autoantibody production and the development of glomerulonephritis. These data showed that Th17/IL-17 is important in lupus pathogenesis (33). Since IL-36 is able to increase the expression of IL-17 in lupus, what is the role of IL-36 in the regulation of Th17/IL-17 signaling? How does IL-36 interact with IL-17 and, therefore, contribute to the development of lupus? Does IL-36 regulate some downstream signaling pathways and then affect the production of IL-17, aggravating lupus development? Hopefully, all these questions will be answered in the future.

### IL-36 in Psoriasis

Psoriasis is a chronic and relapsing inflammatory skin disease. Plaque psoriasis is the most common form of the disease with a worldwide prevalence 1–2%. The main pathological manifestations include excessive epidermal keratinization and parakeratosis, hypertrophy of stratum spinosum cells, lymphocyte infiltration, and vasodilation. Genetic factors, infections, and immune cell dysfunction play important roles in this complex and polygenic autoimmune disease. Epidemiological studies involving twins and families suggest that psoriasis is an inheritable disease. For instance, monozygotic twins had higher concordance of psoriasis than dizygotic twins (34). Variation in the *HLA-C* gene contributes to psoriatic heredity, by which HLA-C^*^06 is related to psoriasis patients in White (35) and Chinese (36) populations. A genome-wide association study identified numerous genetic risk factors associated with psoriasis, such as *LCE3B, LCE3C, CSTA* (involving skin barrier function), *IL12B, IL23A, IL23R, TYK2, IFIH1*, and *ERAP1*, and *ZAP70* (in immunological response) (37). Another study with whole-exome single-nucleotide polymorphism array found that genes such as *C1orf141, ZNF683, TMC6, AIM2, IL1RL1, CASR, SON, ZFYVE16*, and *MTHFR* were related to psoriasis in Chinese (38). These findings indicated that psoriasis is a polygenic disorder. As compared to psoriasis, deficiency of IL-36R antagonist (DITRA) is a life-threatening monogenic type of disease caused by loss-of-function mutations in the *IL36RN* gene. Affected patients suffered from recurrent episodes of generalized pustular psoriasis with skin impairment and systemic inflammation. Several pathogenic variants in the *IL-36RN* gene have been recognized to correlate with DITRA, such as c.41C>A, c.41T>C, and c.420_426del (39). Clinically, patients with DITRA present with symptoms in their childhood or adolescence. Inhibition of TNF-α, IL-12/23, and IL-17 is effective in suppressing the disease activity in patients with DITRA, while anti-IL-1 treatment seems less effective (40). Therefore, DITRA is an example of monogenic IL-36-mediated disease, which is different from psoriasis.

Serum levels of IL-36 in patients with psoriasis were higher than those in healthy controls, and the elevated levels of IL-36 correlated with disease activity (41, 42). A recent study that discussed anti-IL-36 treatment in pustular psoriasis patients showed that the monoclonal antibody against IL-36R reduced symptoms of the disease (43). In imiquimod-induced psoriasis (a psoriasis mouse model), IL-36α injection contributed to the development of psoriasis, especially severe skin lesions (20). Psoriatic mice treated with IL-36R-blocking antibodies showed improved psoriatic dermatitis (14). IL-36 signaling-related genes were upregulated in psoriatic skin lesions and enriched within psoriasis susceptibility loci when human primary keratinocytes were treated with bioactive IL-36 (14). With IL-36α, IL-36β, or IL-36γ stimulation, S100A8, IL-17C expression is increased in wild-type keratinocytes compared to IL-36R^−/−^ keratinocytes (44), suggesting the role of the IL-36/IL-36R autocrine loop within keratinocytes in psoriasis development. *AP1S3* mutations have been widely demonstrated as risk factors in psoriasis. *AP1S3* deficiency enhanced Toll-like receptor (TLR) 2/6 signaling, such as MALP-2, which induced the expression of IL-36α in keratinocytes and impaired keratinocyte autophagy. Furthermore, individuals with psoriasis showed a particularly severe, recalcitrant phenotype that carried both the *IL36RN* and *AP1S3* mutations (24). IL-36 combined with IL-1 to recruit neutrophils in the dermis and epidermis and, then, promoted the inflammatory keratinocyte response via inducing the expression of inflammatory chemokines, such as IL-8 (45), suggesting that the IL-36/IL-1–chemokine–neutrophil axis may have a role in the pathogenesis of psoriasis. In addition, keratinocytes stimulated with IL-36 (IL-36α, IL-36β, and IL-36γ) significantly upregulated CCL20, MMP9, and IRAK2 (14). It has been shown that Th17 cells and related cytokines (IL-23/IL-17/IL-22 axis) participated in the pathogenesis of psoriasis (46). In imiquimod-induced psoriasis mice, IL-36Ra deficiency drove skin lesions and induced IL-23, IL-17, and IL-22 expression, whereas the wild-type mice did not develop skin lesions (46). These findings suggested that IL-36 contributes to the pathogenesis of psoriasis.

### IL-36 in Arthritis

RA is a chronic inflammatory autoimmune disease. The pathological symptom of RA is synovitis of the joints, with the proliferation of synovial cells, the formation of neovascularization, and local infiltration of lymphocytes. IL-36α, IL-36R, and IL-36Ra were detected in the synovial tissues of patients with RA, and the expression of IL-36α in the synovial tissue was higher in RA patients than in osteoarthritis (OA) patients (47). IL-36 stimulation with synovial fibroblasts promoted the production of IL-6 and IL-8 (47). Similarly, IL-36R and IL-36γ were highly expressed in the joints of collagen-induced arthritis mice (48). The limited data indicated that IL-36 may correlate with RA pathogenesis. Spondyloarthritides (SpA) are a group of chronic systemic inflammatory joint diseases, and psoriatic arthritis (PsA) is one of the main types. The expression of IL-36R and its ligands IL-36α and IL-36Ra can be detected in the synovial lining layer and in the cellular infiltrates of patients with PsA (47). IL-36α expression was higher in PsA patients compared with that in OA patients. CD138^+^ plasma cells were the main cellular source of IL-36α, which promoted the generation of IL-6 and IL-8 in fibroblast-like synoviocytes by activating the p38/NF-κB signaling pathways (47). OA is a debilitating disease, and older people are more susceptible to this common disorder. IL-36α/IL-36R expression was increased in OA patients' cartilage compared to controls (49). Mice deficient in TGFBR2 showed an uncavitated, disorganized cluster of cells and abnormal condyle morphology after the destabilization of the medial meniscus (DMM) procedure, whereas wild-type mice developed knee joint cavities containing cartilage, menisci, and supporting ligaments in the knees (49). Similarly, after DMM surgery, wild-type mice showed a decrease in IL-36Ra and an increase in IL-36α, IL-36β, IL-36γ, and IL-36R within the regions of increased mechanical loading, indicating that, in articular cartilage, the abnormal mechanical loading caused by DMM may trigger downregulation of IL-36Ra and upregulation of IL-36/IL-36R to induce OA progression (49). Moreover, after injection of IL-36Ra into the knee joints of TGFBR2^−/−^ mice or DMM-induced OA mice, there was attenuation of OA progression, accompanied with a decrease in chondrocyte hypertrophy, reduced fibrillation, reduced matrix destruction, and reduced expression of collagen 10 and MMP13. It is notable that the injection of IL-36α not only induced an OA-like phenotype in wild-type mice but also worsened OA in TGFBR2^−/−^ mice, suggesting that IL-36α promoted OA development (49).

### IL-36 in IBD

IBD is a chronic intestinal inflammatory disease that encompasses Crohn's disease and ulcerative colitis. The causes of the disease remain unknown. IL-36α and IL-36γ were elevated in mucosal biopsies from patients with IBD (50, 51). In dextran sodium sulfate (DSS)-induced colitis mice, IL-36 administration promoted colon injury and inflammation, and the colonic expression of IL-36α and IL-36γ was markedly elevated. Interestingly, IL-36R^−/−^ mice showed reduced disease progression after treatment with DSS, whereas wild-type mice developed severe symptoms in the presence of DSS (22). CD4^+^ T cells from the mucosa of the colon of mice with colitis treated with IL-36α and IL-36γ showed significant induction of Th1 cell differentiation (22). Furthermore, IL-36α and IL-36γ stimulation induced the expression of chemokines and acute phase proteins in an intestinal epithelial cell line (HT-29 cells) and induced the assembly of MyD88, TRAF6, IRAK1, and TAK1 and the transactivation of NF-κB, AP-1, and MAPK, therefore enhancing inflammation of the colon (52). The above studies showed that IL-36 is involved in the pathogenesis of IBD.

### IL-36 in Other Systemic Inflammatory Diseases

Studies indicated that IL-36 family cytokines have emerged as important inflammatory mediators in different inflammatory autoimmune disorders, such as SpA and primary Sjögren's syndrome (pSS). SpA comprises a group of diseases including ankylosing spondylitis (AS), reactive arthritis (ReA), PsA, and IBD-related arthropathy (53). *IL-38* gene polymorphisms are associated with both AS and PsA (54, 55). Interestingly, IL-38 and IL-36Ra have demonstrated similar anti-inflammatory effects. Previous studies have reported that the pathogenesis of psoriasis is related to the IL-23/IL-17/IL-22 axis. Therefore, it is hypothesized that IL-36 may be involved in the pathogenesis of SpA by increasing the generation of pro-inflammatory cytokines, such as IL-23, IL-17, IL-22, TNF-α, and IL-6, as well as promoting the inflammatory response, similar to the role of IL-38 in SpA. However, further studies are needed to validate this hypothesis. Serum levels of IL-36α were elevated in pSS patients compared with those in healthy controls (56). The expression of IL-36α in the minor salivary glands of pSS patients was significantly higher compared to that in controls. It is notable that αβ^+^CD3^+^ T cells and CD68^+^ cells were the major sources of IL-36α in minor salivary glands of pSS patients (57). In addition, serum levels of IL-36α in autoimmune blistering disease patients were significantly higher than those in controls, and elevated IL-36α was related to disease activity (56) (Table 1).

**Table 1:** Abnormal expression of interleukin (IL)-36 in inflammatory diseases.

**Disorder**	**Expression of IL-36**	**References**
Systemic lupus erythematosus	Serum	(27–30)↑
Psoriasis	Serum	(41, 42)↑
Rheumatoid arthritis	Synovial tissue	(42, 47)↑
Inflammatory bowel disease	Colonic mucosa	(50, 51)↑
Primary Sjögren's syndrome	Serum	(57)↑
Blistering disease	Serum	(56)↑

## Conclusion

IL-36 is a member of the IL-1 superfamily. IL-36 has similarities to IL-1 in its signaling pathway and biochemical function. IL-36 is expressed in a variety of cells and tissues. Keratinocytes are important target cells for IL-36. In recent years, growing evidence has indicated that IL-36 plays important roles in systemic inflammatory diseases. Both animal and *in vitro* experimental studies and patient samples showed the effect of IL-36 in the pathogenesis of these disorders. However, several points should be noted. Mutations in IL-36RN can lead to DITRA, which is correlated with a severe course of pustular psoriasis. The IL-23/IL-17/IL-22 axis, which is induced by IL-36, plays an important role in psoriasis. SLE is a complex autoimmune disease with a poor prognosis. A previous study suggested that the pathogenesis of SLE correlated with abnormal B-cell frequency or function (58). The relationship between IL-36 and B cells is rarely discussed. Therefore, how IL-36 is involved in the pathogenesis of SLE by regulating the dysfunction of B cells warrants further investigation. Since the above findings indicate a significant relationship between IL-36 and disease activity in different inflammatory autoimmune diseases, including SLE, there is great potential for IL-36 as a biomarker for these diseases. To test the potential of IL-36 as a disease activity marker, a specific cohort of patients with preclinical disease, early disease, or long-standing disease, which are situations in which IL-36 has demonstrated diagnostic or predictive utility, should be recruited.

In conclusion, much progress has been made in recent years in studying the role of IL-36 cytokines in the pathogenesis of systemic inflammatory diseases, especially regarding their roles in regulating skin inflammation. The information in this review may aid in guiding the development of novel targeted therapeutic strategies.

## Author Contributions

Z-CY, X-YaL, X-YoL, W-DX, A-FH, and L-CS designed and wrote this paper. All of the co-authors agreed on the submission of the paper.

### Conflict of Interest

The authors declare that the research was conducted in the absence of any commercial or financial relationships that could be construed as a potential conflict of interest.
